# The Art of Nursing as a Part of the Education of Women

**Published:** 1888-03-17

**Authors:** 


### The Nursing Mirror.

Communications for this column should be addressed The Mirror, care of the Editor, 38, Old Jewry, London, E.C.

### THE ART OF NURSING AS A PART OF THE EDUCATION OF WOMEN.

Now that we are daily becoming more enlightened, and learning better to appreciate knowledge in all its various forms, is there any branch of it which we should regard as more essential to the welfare of humanity than knowing how to nurse our fellow-creatures in time of sickness? There are plenty of women now seeking to make themselves proficient in the art of nursing, and taking up the vocation as a profession; but I wish to suggest that young women in private life should be taught how to nurse, so that they would be of use should illness suddenly enter their homes; there being numerous occasions when a trained nurse cannot be obtained at a minute's notice, or, again, when the illness may not be deemed so important as to necessitate the aid of one, yet at the same time may need careful and intelligent nursing. It is these instances I am more especially considering, it being obvious that in surgical cases, or where the illness becomes of a serious nature, the presence of a thoroughly-trained nurse is not only desirable but essential. There are doubtless hundreds of women who would be ready to do their best when called upon to act as nurses to those they love, but it is doubtful how many of them would be of any real assistance in a sick room, not from want of will, but from want of the necessary knowledge. The knowledge, for example, how to make a poultice, or how to prepare a cup of gruel or arrowroot—things which sound simple enough in themselves, yet requiring practice; for a poultice unless rightly made is useless, or worse than useless, and a cup of gruel or arrowroot which is not properly prepared would neither be palatable nor calculated to do much good to the patient. It may possibly be contended that the making of arrowroot or gruel should devolve on the duties of one's own cook; but supposing one to be away from home, and perhaps at the mercy of the maid-of-all-work in a lodging-house, where the making of invalid food is seldom properly understood, how essential it then becomes for a woman to be able to attend to these small but important trifles herself. Parents in having their daughters taught foreign languages, music, and painting, do not necessarily do so with a view to their becoming professional pianists and painters and teachers of languages, but that these additions to their education should prove a source of pleasure and use to them through life. And with a like view I maintain that a woman should be taught those requirements which would enable her to tend the sick properly, apart from any idea of her taking up nursing as a profession; for then, when suddenly called upon to act, she will feel herself as much at home in the sick room, making poultices, preparing invalid food, and fulfilling the various duties which need not here be enumerated, as she would be in her drawing-room performing on the piano, painting, or reading books in foreign languages; and I consider that this addition to the education of our daughters should be looked upon as being of as much importance as the other branches of learning. It is a knowledge which can only tend towards making a woman that which it should be the aim of every true woman to become —viz., a being of use to her fellow-creatures, and *able* as well as *willing* to serve them in a time of need. A. R. A

